# Effect of high-risk sleep apnea on treatment-response to a tailored digital cognitive behavioral therapy for insomnia program: a quasi-experimental trial

**DOI:** 10.3389/frsle.2024.1355468

**Published:** 2024-03-13

**Authors:** Alexander Sweetman, Chelsea Reynolds, Leon Lack, Andrew Vakulin, Ching Li Chai-Coetzer, Douglas M. Wallace, Megan Crawford, Cele Richardson

**Affiliations:** ^1^Adelaide Institute for Sleep Health and Flinders Health and Medical Research Institute: Sleep Health, College of Medicine and Public Health, Flinders University, Adelaide, SA, Australia; ^2^College of Education, Psychology and Social Work, Flinders University, Adelaide, SA, Australia; ^3^Medicine Service, Bruce W. Carter Department of Veterans Affairs Medical Center, Miami, FL, United States; ^4^Department of Neurology, Sleep Medicine, University of Miami Miller School of Medicine, Miami, FL, United States; ^5^Department of Psychological Sciences and Health, Strathclyde Centre for Sleep Health, University of Strathclyde, Glasgow, United Kingdom; ^6^School of Psychological Science, University of Western Australia, Perth, WA, Australia

**Keywords:** difficulties initiating and maintaining sleep, non-pharmacological, sleep disordered breathing, clinical trial, insomia

## Abstract

**Introduction:**

Therapist-delivered Cognitive Behavioral Therapy for Insomnia (CBTi) is an effective but largely inaccessible treatment for people with Co-Morbid Insomnia and Sleep Apnea (COMISA). To increase CBTi access for COMISA, we aimed to develop a self-guided interactive 5-session digital CBTi program that is appropriate for people with insomnia-alone and COMISA, and compare its effectiveness between people with insomnia-alone, vs. comorbid insomnia and high-risk sleep apnea.

**Methods:**

Data from 62 adults with insomnia symptoms were used. High-risk sleep apnea was defined as a score of ≥5 on the OSA50. Participants self-reported symptoms of insomnia (ISI), depression, anxiety, sleepiness (ESS), fatigue, and maladaptive sleep-related beliefs (DBAS-16) at baseline, 8-week, and 16-week follow-up. ESS scores were additionally assessed during each CBTi session. Intent-to-treat mixed models and complete-case chi^2^ analyses were used.

**Results:**

There were more participants with insomnia-alone [*n* = 43, age *M* (sd) = 51.8 (17.0), 86.1% female] than suspected COMISA [*n* = 19, age = 54.0 (14.8), 73.7% female]. There were no between-group differences in baseline questionnaire data, or rates of missing follow-up data. There were no significant group by time interactions on any outcomes. Main effects of time indicated moderate-to-large and sustained improvements in insomnia (*d* = 3.3), depression (*d* = 1.2), anxiety (*d* = 0.6), ESS (*d* = 0.5), fatigue (*d* = 1.2), and DBAS-16 symptoms (*d* = 1.2) at 16-weeks. ESS scores did not increase significantly during any CBTi session.

**Conclusion:**

This interactive digital CBTi program is effective in people with insomnia-alone, and people with co-morbid insomnia and high-risk sleep apnea. Further research is required to determine the effectiveness, safety and acceptability of digital CBTi in people with insomnia and confirmed sleep apnea.

**Clinical Trial Registration:**

This trial was prospectively registered on the Australian and New Zealand Clinical Trials Registry (ANZCTR, ACTRN12621001395820).

## 1 Introduction

Insomnia and obstructive sleep apnea (OSA) are the two most prevalent sleep disorders and frequently co-occur (AASM, 2014; Sweetman et al., 2017a). Approximately 30–40% of people with insomnia have co-morbid OSA (Luyster et al., 2010; Sweetman et al., 2021), however most people with OSA remain undiagnosed (Lichstein et al., 1999). People with Co-Morbid Insomnia and Sleep Apnea (COMISA) generally have worse sleep (Bianchi et al., 2013), daytime function (Krakow et al., 2001), mental health (Lang et al., 2017), physical health (Lechat et al., 2022a), productivity (Sivertsen et al., 2013), and quality of life (Björnsdóttir et al., 2015) compared to people with neither sleep disorder, and often compared to people with either insomnia-alone or OSA-alone (Sweetman et al., 2019a; Ong et al., 2020a). Three recent population-based studies have reported that people with COMISA experience a 50–70% increased risk of mortality over 10–20 years of follow-up, compared to people with neither condition (Lechat et al., 2021, 2022b; Sweetman et al., 2022). Given the high prevalence and adverse health consequences of COMISA, it is important to develop and implement effective evidence-based management approaches for this condition (Sweetman et al., 2017a).

Cognitive Behavioral Therapy for insomnia (CBTi) is the recommended “first line” treatment for insomnia (Qaseem et al., 2016; Ree et al., 2017; ASA, 2024), and is effective in patients with COMISA (Sweetman et al., 2023a). CBTi is a multi-component therapy that has historically been delivered by trained therapists/psychologists over 4–8 weekly sessions. CBTi aims to identify and treat the underlying precipitating triggers and perpetuating factors that maintain insomnia, and leads to sustained improvements in sleep (Morin et al., 1994; van der Zweerde et al., 2019a), daytime function and mental health (Ye et al., 2015). A recent systematic review and meta-analysis reported that CBTi is associated with large improvements in insomnia severity in the presence of treated and untreated co-morbid OSA (Sweetman et al., 2023a). Furthermore, CBTi may improve the severity of un-treated OSA (Sweetman et al., 2020a), and two of four recent randomized controlled trials indicate that CBTi may improve subsequent acceptance and use of CPAP therapy in patients with COMISA (Bjorvatn et al., 2018; Sweetman et al., 2019b; Ong et al., 2020b; Alessi et al., 2021).

Despite a wealth of evidence supporting the effectiveness of CBTi, access to this “first line” treatment is extremely limited. In Australia, ~90% of primary care patients with insomnia are managed with sleeping pills while only 1% are referred to psychologists for CBTi (Miller et al., 2017; Haycock et al., 2022). In a recent analysis of the Veterans Health Administration, the largest healthcare system in the US, veterans initially presenting to various clinics with insomnia disorder were 11-times more likely to receive a medication prescription first than to be referred to CBTi (Pfeiffer et al., 2023). In this analysis including over five million veterans, CBTi was provided to only 0.2% of the sample. Rates of access to CBTi across Europe mirror those elsewhere in the world with only 50–3,000 patients per country receiving psychological treatment for their insomnia per year (Baglioni et al., 2020).

Clinician-delivered CBTi programs have been translated to self-guided digital programs which have the potential to increase CBTi availability and access (Thorndike et al., 2008; Espie et al., 2012). However, self-guided digital CBTi programs have generally been developed for people with insomnia-alone, and there is very limited evidence on the effectiveness, safety, or suitability of digital CBTi in people with COMISA (Eldridge-Smith et al., 2022). In particular, people with COMISA may commence treatment with higher levels of daytime sleepiness, be less responsive to self-guided sleep restriction therapy in the presence of apnea events that promote repeated awakenings, or experience increased sleepiness during the acute phase of sleep restriction (Sweetman et al., 2020b; Turner et al., 2023). Because of these concerns, some self-guided digital programs tailored for people with insomnia-alone may not be appropriate for individuals with OSA. Indeed, clinical trials of digital CBTi often exclude people with untreated OSA (Christensen et al., 2016; Ritterband et al., 2017; Nazem et al., 2023) and suspected OSA (Espie et al., 2019; van der Zweerde et al., 2019b; Vedaa et al., 2020). This may fuel the belief that insomnia is “secondary” to OSA, or that OSA must be diagnosed and sufficiently treated before commencing insomnia management (Sweetman et al., 2017a, 2021). However, our systematic review demonstrates that CBTi can be safely and effectively delivered by clinicians to patients with untreated OSA (Sweetman et al., 2023a). These results are particularly welcoming when considering the long delay to diagnosis and treatment of OSA in many countries.

Overnight polysomnography is the “gold standard” measure of OSA presence and severity, however, is costly, time consuming, and often incurs long waiting lists. In the absence of overnight sleep studies, self-report measures have been developed and validated to screen for a “high-risk” of OSA (Chai-Coetzer et al., 2011; Chung et al., 2016). To expedite access to digital CBTi in patients with COMISA, it may be possible to identify people with insomnia and a “high risk” of co-morbid OSA according to self-report insomnia and OSA-screening questionnaires. CBTi could then be administered concurrent to the diagnosis and treatment of possible OSA. However, there are safety concerns when providing sleep restriction therapy to people with suspected COMISA, as this treatment may increase an already higher level of daytime sleepiness (Sweetman et al., 2020b). A digital CBTi program that can tailor therapy to mitigate excessive sleepiness during CBTi would thus be invaluable to patients with suspected COMISA, who otherwise experience very limited access to CBTi partly due to sleepiness-related and safety concerns.

The aim of this study was therefore to develop and test a self-guided digital CBTi program that includes treatment algorithms designed for people with insomnia-alone and COMISA (Sweetman et al., 2023c). We aimed to compare the effectiveness of an interactive digital CBTi program, Bedtime Window, on changes in symptoms of insomnia, daytime function and mental health in people with insomnia-alone vs. people with comorbid insomnia and a “high-risk” of OSA (suspected COMISA group), and monitor weekly changes in sleepiness during treatment.

## 2 Methods

We report here secondary data analysis from an online clinical trial of digital CBTi in a community-based sample of people with insomnia symptoms (Sweetman et al., 2023c). The trial was approved by the Southern Adelaide Clinical Human Research Ethics Committee and registered on the Australian and New Zealand Clinical Trials Registry (ACTRN12621001395820). Secondary data analysis was conducted to investigate the effect of insomnia-alone vs. insomnia and high-risk OSA on treatment-response to digital CBTi from baseline to 8- and 16-week follow-up, and between-group changes in weekly self-reported sleepiness during CBTi.

### 2.1 Participants

People throughout Australia were directed by online, print, radio, and television advertisements to an online information and consent form and questionnaire battery. Participants were assessed for eligibility before being randomized 1:1 to immediate or waitlist (sleep education control) digital CBTi. Eligible participants were contacted via email to inform them of study recruitment and group allocation.

Inclusion criteria were age ≥ 18, reliable access to an internet-compatible device, basic English language comprehension (for access to the intervention), and an Insomnia Severity Index (ISI) score of ≥15 (at least moderate insomnia). Exclusion criteria were bipolar or schizophrenia disorder, risk of suicide or self-harm on the Patient Health Questionnaire-9 (PHQ-9), epilepsy, confirmed co-morbid sleep disorder other than insomnia, doctor diagnosed cognitive impairment, current pregnancy, Epworth Sleepiness Scale (ESS) ≥ 16, people that were drivers for work, shift workers, and people that had experienced a sleepiness-related motor vehicle accident. Ineligible participants were directed to local or tele-health clinical insomnia services.

Participants with a previous doctor diagnosis of OSA were excluded from this study which intended to investigate the effect of a high-risk of undiagnosed (and consequently, untreated) OSA on treatment-response to digital CBTi.

### 2.2 High risk OSA

Participants were included in the suspected COMISA group if they reported a score of ≥5 on the OSA50 (Chai-Coetzer et al., 2011). The OSA50 is a 4-item self-report questionnaire designed to identify people with a high-risk of moderate-to-severe OSA according to the presence of obesity, snoring, witnessed apnea events, and age ≥ 50 years. Body Mass Index (BMI) was used as an indicator of obesity in the absence of waist circumference data (Item 1, BMI Thresholds: Male ≥ 30, Female ≥ 28). Participants with a score of <5 were categorized with insomnia-alone.

### 2.3 Intervention

A five-session self-guided interactive digital CBTi program (Bedtime Window) was used. The program is designed for people with insomnia-alone and COMISA. Each weekly session lasts for ~20–30 min and included short videos, images and text-based information. An initial assessment module is designed to identify patients with insomnia-alone, in addition to those with confirmed or suspected OSA (according to self-reported symptoms indicative of OSA). Treatment components include tailored psychoeducation, stimulus control therapy, sleep restriction therapy, relaxation therapy, cognitive therapy, and sleep hygiene information. Users complete a digital sleep-wake diary on each morning throughout the program, and provide additional lifestyle, sociodemographic, and sleep-wake information during the program which is used to inform automated personalized treatment recommendations. Algorithms tailor therapy recommendations according to sleep, wake and lifestyle parameters reported at baseline and during each weekly session, with several billion unique treatment pathways available to users. People with COMISA may experience greater sleepiness-related risks during CBTi (Sweetman et al., 2020b; Turner et al., 2023). To mitigate these risks in people with COMISA, the program includes algorithms that continuously assess for symptoms of sleepiness and alertness, and provides tailored and interactive recommendations to enact sleep restriction, sleep regularization, sleep compression, and sleep extension therapies based on these data. Immediately following each session, users receive an email with session-specific content and tailored treatment recommendations.

### 2.4 Questionnaire battery

A questionnaire battery including the ISI (Bastien and Vallières, 2001) [further separated into a ISI nocturnal sub-score (first three items), and ISI daytime sub-score (final four items) (Wallace and Wohlgemuth, 2019)], ESS (Johns, 1991), Flinders Fatigue Scale (FFS) (Gradisar et al., 2007), PHQ-9 (Kroenke et al., 2003), Generalized Anxiety Disorder questionnaire (GAD-7) (Spitzer et al., 2006), and Dysfunctional Beliefs and Attitudes about Sleep scale (DBAS-16) (Morin and Vallières, 2007) was completed at each follow-up occasion.

### 2.5 Statistical analyses

SPSS (IBM Statistics, version 28) was used to analyze data. Data are presented as mean and standard deviation/95% confidence intervals for continuous data, and count and proportions for categorical data. Alpha levels of <0.05 were used to infer statistical significance. Intention to treat analyses of changes in questionnaire measures of insomnia symptoms from baseline to 8- and 16-week follow-up between the insomnia-alone and suspected COMISA groups were investigated with linear mixed models. Overall interaction effects were required before investigating Bonferroni-corrected pairwise comparisons. Cohen's *d*-values with pooled baseline standard deviations were used as a standardized measure of effect size. Complete case Chi^2^ analyses were used to analyze between-group responder data, with Cohen's *d*-values reported as standardized measures of effect size (Wilson, 2017). Complete-case between-group differences in insomnia remission (ISI <8), and rates of no/mild insomnia (ISI <15) were investigated at 8- and 16-week follow-up.

To increase statistical power, data from an intervention and waitlist control group of the original randomized controlled trial were collapsed and anchored to the timing that they received CBTi. In the original trial, the waitlist group received digital sleep education (control) between baseline and 8-week follow-up, which had no significant or clinically meaningful effect on any questionnaire outcome (Sweetman et al., 2023c). Immediately after the 8-week follow-up, the waitlist group were provided access to digital CBTi. Therefore, intervention group data at baseline, 8-weeks (post-CBTi), and 16-weeks (long-term follow up) was matched and combined with control data at baseline, 16-weeks (post-CBTi) and 24-weeks (long-term follow-up) for the current study (Sweetman et al., 2023c).

## 3 Results

### 3.1 Participant recruitment, retention, and program completion

In total, 117 participants were screened, of whom 22 with subthreshold insomnia (ISI <15) were directed to a digital CBTi trial in primary care, and 8 with confirmed/treated OSA were directed to a digital CBTi trial in people with confirmed COMISA. Of the remaining 87 participants, 25 were excluded based on self-reported self-harm or suicide risk (*n* = 11), excessive daytime sleepiness (*n* = 5), restless legs syndrome (*n* = 4), current shift work (*n* = 3), doctor-diagnosed cognitive impairment (*n* = 1), narcolepsy (*n* = 1), doctor-diagnosed circadian rhythm disorder (*n* = 1), previous motor-vehicle accident (*n* = 1), and bipolar disorder (*n* = 1). In total, 62 participants were eligible and recruited to the present trial (Table 1).

**Table 1:** Average mean (standard deviation) between-group baseline sociodemographic and insomnia information.

	**All (*n* = 62)**	**Insomnia-alone (*n* = 43)**	**Suspected COMISA (*n* = 19)**	**Between-group *p***
Age, y	52.46 (16.26)	51.80 (16.99)	53.97 (14.82)	0.632
Female, *n* (%)	51 (82.2%)	37 (86.1%)	14 (73.7%)	0.240
Body mass index	25.18 (4.26)	24.26 (3.60)	27.26 (4.98)	**0.026**
Weight (kilograms)	71.21 (14.43)	68.37 (11.56)	77.63 (18.19)	**0.019**
Height (centimeters)	167.94 (8.12)	167.81 (7.95)	168.21 (8.72)	0.861
Insomnia Severity Index	18.60 (2.89)	18.93 (2.85)	17.84 (2.93)	0.174
ISI Nocturnal sub-score	7.15 (1.59)	7.23 (1.63)	6.75 (1.51)	0.519
ISI Daytime sub-score	11.45 (2.33)	11.70 (2.21)	10.90 (2.56)	0.214
Epworth Sleepiness Scale	5.31 (3.84)	4.88 (3.72)	6.26 (4.03)	0.195
Flinders Fatigue Scale	18.29 (6.12)	18.42 (5.78)	18.00 (6.99)	0.806
Patient Health Questionnaire	9.97 (4.74)	9.44 (4.66)	11.16 (4.82)	0.191
Generalized Anxiety Disorder scale	7.26 (5.12)	7.49 (5.31)	6.74 (4.75)	0.598
Dysfunctional Beliefs and Attitudes about Sleep scale	58.83 (13.33)	59.67 (14.96)	56.94 (8.65)	0.370
Current sleeping pill use, *n* (%)	30 (48.4%)	22 (51.1%)	8 (42.1%)	0.511

Bold text indicates statistical significance.

There was no difference in the prevalence of suspected COMISA between the intervention (32.2%) and control groups of the original randomized controlled trial (29%, *p* = 0.78). After collapsing and time-matching the intervention and control groups, there were no differences between the insomnia-alone and COMISA groups in rates of missing data at 8-weeks [post-intervention 6 (14%), vs. 6 (31.6%), respectively, *p* = 0.11] or 16-weeks [follow-up assessment 8 (18.6%), vs. 6 (31.6%), respectively, *p* = 0.26]. There were no significant differences between the insomnia-alone and suspected COMISA groups in completion rates of Sessions 1 (86.0 vs. 78.9%), 2 (76.7 vs. 68.4%), 3 (72.1 vs. 68.4%), 4 (72.1 vs. 68.4%), or 5 (full program completion 65.1 vs. 68.4%, all Chi^2^ *p* > 0.05).

### 3.2 Baseline information

Sample characteristics and between-group differences in baseline characteristics are presented in Table 1. The suspected COMISA group had higher average BMI, and greater weight (kilograms) than the insomnia-alone group. There were no other between-group differences in sociodemographic variables or questionnaire data at baseline.

### 3.3 Effectiveness of digital CBTi in patients with insomnia vs. high-risk OSA

**Figure 1:**
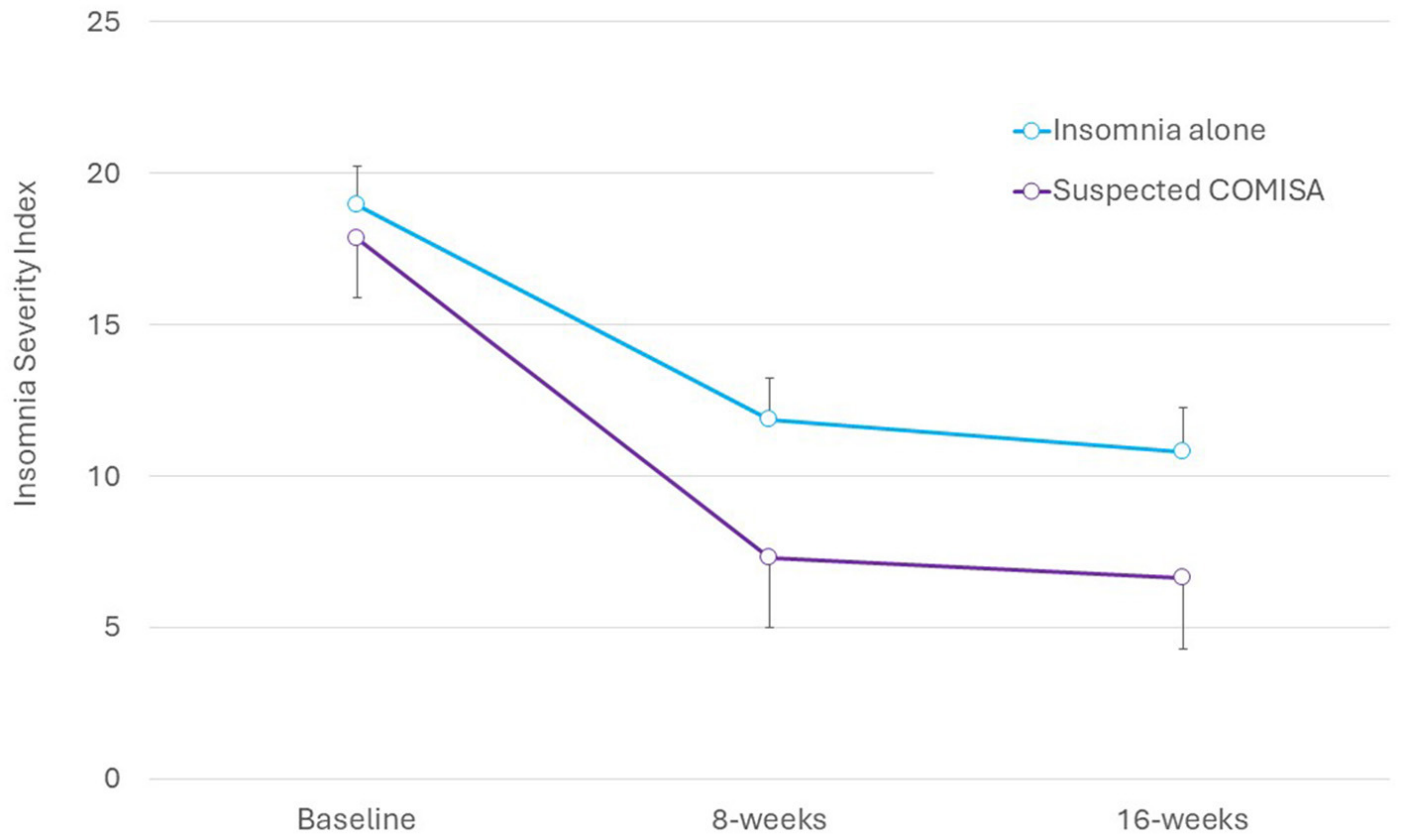
Between-group changes in mean (95% CI) insomnia severity index scores.

There were no significant group by time interactions on the ISI (Figure 1), Flinders Fatigue Scale, ESS, PHQ-9, GAD-7, or DBAS-16 (all interaction *p* > 0.06, Table 2). Main effects of time indicated moderate-to-large and statistically significant improvements in all questionnaire outcomes by 16-week follow-up (Table 2). For the overall sample, there was a large improvement in ISI scores from baseline to 8-weeks (*M* reduction = 7.09, 95% CI = 5.16–9.02, *d* = 3.05, *p* < 0.001), and no subsequent change from 8- to 16-weeks (*M* reduction = 1.02, 95% CI = −0.99 to 3.03, *d* = 0.29, *p* = 0.657). Surprisingly, mean scores indicated that improvements in insomnia, sleepiness, and depression symptoms tended to be larger (although not statistically significant) in the suspected COMISA than insomnia-alone group (Table 2).

**Table 2:** Between-group changes in estimated marginal mean (95% CI) insomnia symptoms during treatment.

	**Baseline**		**8-weeks**		**16-weeks**		**Main time effect *p***	**Main time effect *d***	**Interaction**
	**Insomnia-alone**	**Suspected COMISA**	**Insomnia-alone**	**Suspected COMISA**	**Insomnia-alone**	**Suspected COMISA**			
Insomnia Severity Index	18.93 (1.31)	17.84 (1.96)	11.84 (1.39)	7.31 (2.33)	10.82 (1.44)	6.64 (2.37)	<0.001	3.3	0.066
ISI Nocturnal sub-score	7.23 (0.64)	6.95 (0.97)	4.77 (0.68)	3.21 (1.11)	4.61 (0.70)	3.41 (1.15)	<0.001	1.9	0.136
ISI Daytime sub-score	11.70 (0.86)	10.89 (1.30)	7.08 (0.92)	4.12 (1.54)	6.19 (0.95)	3.24 (1.56)	<0.001	2.8	0.079
Epworth Sleepiness Scale	4.88 (1.16)	6.26 (1.74)	4.71 (1.21)	4.83 (1.96)	4.64 (1.25)	2.94 (2.05)	0.026	0.5	0.062
Flinders Fatigue Scale	18.42 (1.87)	18.00 (2.81)	14.34 (1.98)	11.31 (3.26)	13.36 (2.05)	8.85 (3.36)	<0.001	1.2	0.203
Patient Health Questionnaire	9.44 (1.42)	11.16 (2.14)	6.52 (1.50)	5.24 (2.47)	5.99 (1.56)	3.66 (2.56)	<0.001	1.2	0.053
Generalized Anxiety Disorder questionnaire	7.49 (1.47)	6.74 (2.21)	5.23 (1.54)	4.00 (2.50)	5.38 (1.59)	2.81 (2.61)	<0.001	0.6	0.518
Dysfunctional Beliefs and Attitudes about Sleep	59.67 (5.02)	56.94 (7.54)	49.68 (5.23)	39.61 (8.43)	49.48 (5.41)	35.38 (8.81)	<0.001	1.2	0.101

Responder analyses (Table 3) were undertaken to investigate the between-group proportion of participants that reported insomnia remission (ISI <8), and no/mild insomnia (ISI <15) at 8- and 16-week follow-up. Participants were included if they completed the 8- and 16-week assessments, respectively (irrespective of whether they accessed/completed the intervention). Those with suspected COMISA were more likely to report insomnia remission at the 16-week follow-up compared to those with insomnia-alone. There were no other between-group differences in insomnia response rates at 8 or 16-week follow-up.

**Table 3:** Between-group differences in insomnia response rates at 8-week and 16-week follow-up.

	**Insomnia-alone**	**Suspected COMISA**	**Chi2 *p***	**Cohen's *d***
**8-week follow-up**				
ISI <8	11/37 (29.7%)	5/13 (38.5%)	0.562	0.29
ISI <15	26/37 (70.3%)	12/13 (92.3%)	0.110	0.90
**16-week follow-up**				
ISI <8	9/35 (25.7%)	8/13 (61.5%)	**0.021**	0.84
ISI <15	26/35 (74.3%)	12/13 (92.3%)	0.172	0.79

Cohen's *d* calculated according to Wilson (2017).

### 3.4 Effect of digital CBTi on weekly daytime sleepiness

**Figure 2:**
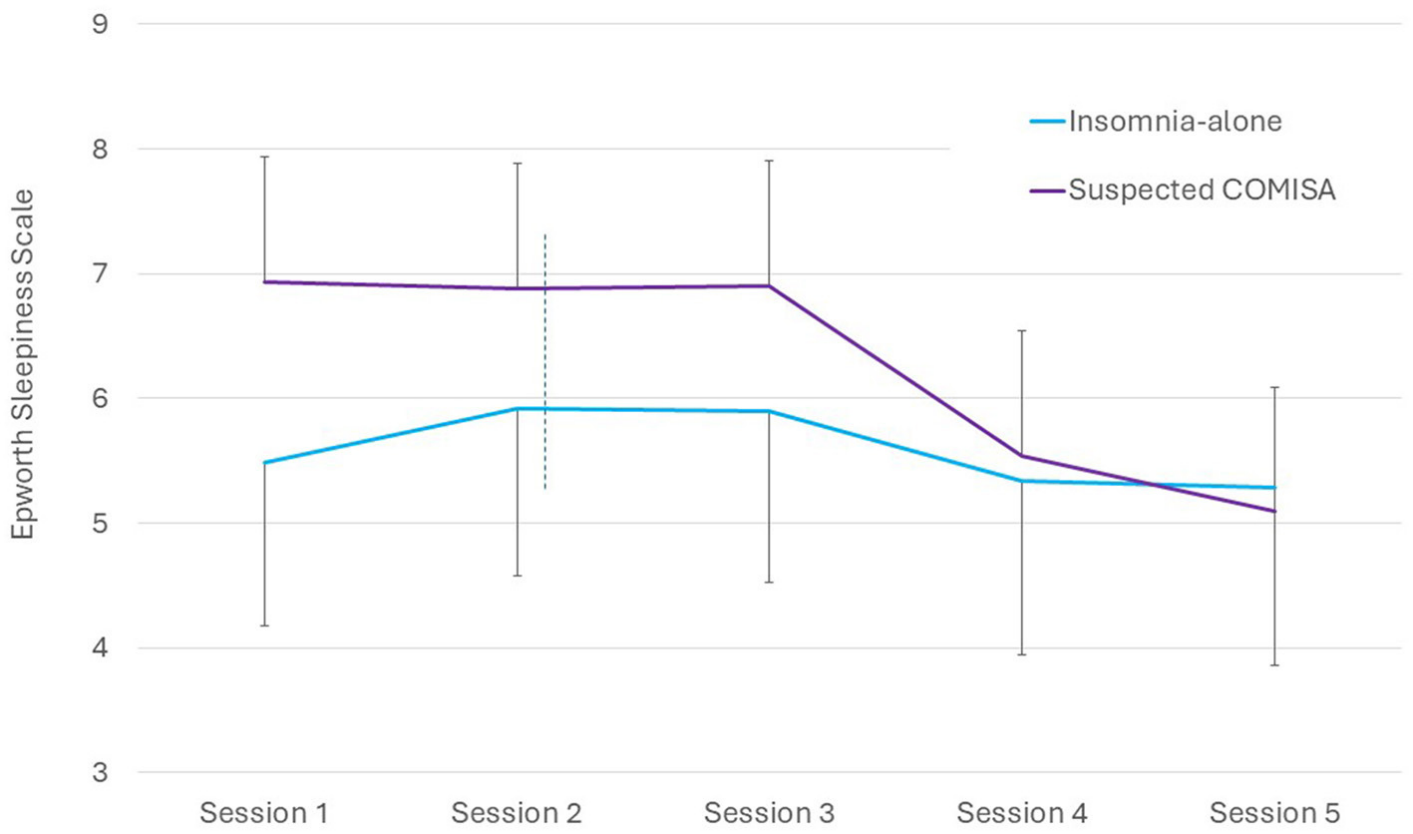
Between-group changes in mean (95%CI) Epworth Sleepiness Scale scores during each weekly digital CBTi session. Dashed line indicates commencement of personalized sleep restriction therapy during Session 2.

The ESS was administered during each weekly session of the CBTi program. There were no between-group differences in changes in ESS scores from baseline to any weekly session (interaction *p* = 0.764, Figure 2). Main effects of time indicated no significant change in ESS scores during the five-sessions of the program (*p* = 0.191). However, as mentioned above, a main effect of time indicated a reduction in ESS scores from baseline to 16-week follow-up (Table 2).

## 4 Discussion

The main finding of this study is that this interactive digital CBTi program was associated with large and sustained improvements in nocturnal and daytime symptoms of insomnia and associated mental health symptoms in participants with insomnia-alone, and in those with a high-risk of undiagnosed OSA. The lack of any significant group by time interactions provides preliminary evidence to suggest that those with high-risk of OSA benefited from this digital CBTi program at least as much as those with insomnia-alone. An important secondary finding is that symptoms of daytime sleepiness did not increase significantly during any week of the digital CBTi program in either group, suggesting that the program may be an effective, safe, and scalable treatment for insomnia in people with suspected COMISA. Importantly, further research in patients with a doctor diagnosis of OSA according to gold-standard polysomnography is required to confirm these promising initial results, before scaling access in different settings in which patients with COMISA are managed.

A non-significant trend was observed that indicated greater overall improvements in symptoms of insomnia, sleepiness and depression in the suspected COMISA group compared to the insomnia-alone group. Caution should be applied in interpreting these non-significant interaction effects, which were of limited clinical significance, and may have also been inflated by the multiple repeated-measures outcomes that were analyzed. The between-group difference in rates of insomnia remission at 16-weeks should similarly be interpreted with caution, given that these “responder” outcomes were based on complete-case analyses, and a small overall sample was recruited. Of more importance, we observed no evidence that participants with a high risk of co-morbid OSA experienced a *reduced* treatment-response to this digital CBTi program compared to those with insomnia-alone. Indeed, the most striking findings were the main effects of time that indicated moderate-to-large and sustained improvements in all outcomes for both groups combined.

In a recent systematic review and meta-analysis of the effect of CBTi in people with COMISA (Sweetman et al., 2023a), we observed a large effect of CBTi on improving ISI scores in people with insomnia and co-morbid treated and un-treated OSA. In the present study, those in the suspected COMISA group also experienced a large and sustained 11.2-point average ISI improvement by 16-weeks. These data suggest that CBTi is an efficacious treatment for insomnia in the presence of high-risk co-morbid OSA, when delivered by trained clinicians, or via an interactive self-guided digital program tailored for COMISA. Before scaling access to self-guided digital CBTi in people with COMISA, these results require replication in larger samples of patients with baseline sleep studies to confirm the presence and severity of co-morbid sleep apnea. One additional pilot study is currently being conducted in people with co-morbid insomnia and a doctor-diagnosis of OSA (Sweetman et al., 2022), and one study is being conducted in people with insomnia-alone and COMISA in primary care (Sweetman et al., 2022). Additional research is also required to determine the effectiveness and acceptability of digital CBTi in patients with confirmed COMISA recruited from different settings, including sleep clinics in which patients may present with more severe OSA, symptomatic OSA (i.e., with higher levels of sleepiness at baseline), and different factors that motivate treatment-seeking and engagement (Sweetman et al., 2019b; Alessi et al., 2021).

Although self-guided digital CBTi programs have been studied in samples with insomnia (Soh et al., 2020), and co-morbid insomnia and mental health symptoms (Ye et al., 2015), there has been understandable reservation about using self-guided digital CBTi in patients with COMISA (Eldridge-Smith et al., 2022). Sleep restriction therapy is one of the most effective behavioral components of CBTi, which aims to temporarily reduce time in bed, consolidate sleep periods and reduce time awake throughout the night (Spielman and Saskin, 1987). Similarly, stimulus control therapy which aims to re-associate the bed with a state of sleep and rest rather than conditioned arousal, requires the patient to “get out of bed if not asleep within about 15 minutes”, and consequently may delay sleep onset and reduce sleep duration in the early treatment phase (Bootzin, 1972). Although these behavioral treatments often lead to rapid improvement in insomnia, they are associated with an acute increase in daytime sleepiness in patients with insomnia alone (Kyle et al., 2014) and COMISA (Sweetman et al., 2020b). This increase in sleepiness is reduced after sleep improves, and time in bed is gradually extended from week to week until a comfortable equilibrium between sleep duration, time in bed, and sleepiness is achieved (Sweetman et al., 2020b). However, patients with COMISA may commence treatment with pre-existing levels of sleepiness due to un-treated OSA (Sweetman et al., 2017b), and experience an increased vulnerability to the effects of sleep restriction on daytime sleepiness and neurocognitive function (Vakulin et al., 2009; Turner et al., 2023). Hence, there has been reservation about the use of digital CBTi programs designed for patients with insomnia-alone, in patients with COMISA. In the current study, participants with insomnia-alone and suspected COMISA did not report any overall increase in daytime sleepiness during any week of the digital CBTi program. This is likely due to specific algorithms within the program that continuously monitor for levels of alertness and sleepiness, and adapt personalized therapy recommendations to mitigate risk of alertness-failure. In fact, an overall effect of time on reduced levels of daytime sleepiness was observed by 16-week follow-up. Given reports that CBTi is associated with acute impairment in neurocognitive function (Turner et al., 2023), future research is required to confirm the safety of this digital CBTi program in patients with different levels of OSA severity, and in the presence of excessive daytime sleepiness, before implementing broader access.

The recommended “first line” treatment for moderate and severe OSA is Continuous Positive Airway Pressure (CPAP) therapy, and lifestyle/weight management recommendations where indicated (Epstein et al., 2009; ASA, 2024). Although CPAP therapy improves stability of the upper airway during sleep and many of the associated consequences of OSA, it is limited by suboptimal acceptance and long-term use for the duration of the sleep period (Weaver and Grunstein, 2008). Co-morbid insomnia symptoms are associated with approximately a 30% reduction in initial acceptance of CPAP therapy, and a 2-h reduction in average nightly CPAP use compared to patients with OSA alone (Sweetman et al., 2023b). Two of four recent randomized controlled trials have reported that CBTi improves subsequent acceptance and use of CPAP therapy in patients with COMISA (Bjorvatn et al., 2018; Sweetman et al., 2019b; Ong et al., 2020b; Alessi et al., 2021). An ongoing study aims to investigate the effect of digital CBTi on insomnia symptoms and CPAP use among people with COMISA after commencement of CPAP recommendations and management (Eldridge-Smith et al., 2022). Future studies should also investigate the possibility of using digital CBTi to treat insomnia prior commencing CPAP, to improve initial experiences with CPAP therapy (i.e., the first week of use), and improve long-term CPAP use in patients with confirmed COMISA.

### 4.1 Limitations

Although this study has several strengths including standardized questionnaire measures of high-risk OSA and insomnia symptoms, acceptable rates of digital CBTi completion (65–68%), and a low rate of missing 16-week follow-up data for an online trial (22.5%), there are several important limitations.

Firstly, COMISA was defined according to self-reported symptoms of insomnia and OSA in the absence of a clinician diagnosis or overnight sleep studies. Overnight polysomnography is the gold-standard measure of OSA presence and severity, and will be required in future clinical trials investigating the safety and effectiveness of digital CBTi in people with COMISA. Although the OSA50 is a standardized measure with good sensitivity to indicate a high-risk of moderate-severe OSA (Chai-Coetzer et al., 2011), specificity levels of ~50% mean that half of those in the “COMISA” group may have no/mild OSA. Hence, this study provides promising initial evidence of the effectiveness of this digital CBTi program in those with a high-risk of OSA. However due to the risk of false-negative and false-positive OSA cases identified according to the OSA50, these results will need to be confirmed in future clinical trials in participants with confirmed OSA (Sweetman, 2022a,b). Two randomized controlled trials investigating the effectiveness of Bedtime Window in people with confirmed COMISA are presently being conducted.

Secondly, the sample may not be reflective of patients with COMISA in sleep clinic settings, and consequently, results may not directly generalize. For example, the majority of participants were female, had low levels of daytime sleepiness, and moderate insomnia severity. Although the COMISA group had a BMI in the “overweight” range, which was higher than those with insomnia-alone, it was still lower than average BMI of participants with COMISA in several previous clinical trials (Ong et al., 2017; Bjorvatn et al., 2018; Sweetman et al., 2019b). Future trials are required to implement and understand the generalizability of this digital CBTi program in improving insomnia among people with COMISA in different settings.

Third, no information on neurocognitive functioning was collected. Clinician-delivered CBTi may be associated with an acute reduction in neurocognitive functioning (Turner et al., 2023), likely due to sleep restriction therapy resulting in acute sleep loss, before conditioned insomnia is reduced and sleep improves (Kyle et al., 2014). This digital CBTi program was not associated with increased daytime sleepiness during any weekly session which is promising given previous research reporting an increase in daytime sleepiness during clinician-delivered CBTi in people with insomnia (Kyle et al., 2014) and COMISA (Sweetman et al., 2020b). However, the ESS may not be reflective of objective sleepiness in people with OSA, and additional research is required to investigate changes in more sensitive objective measures of alertness and neurocognitive function during digital CBTi in patients with COMISA.

Finally, results of responder analyses should be interpreted with caution, given that they are based on participants with observed follow-up data. There were no between-group differences in rates of missing data at 8- or 16-week follow-up, and overall rates of missing data were relatively low for a clinical trial conducted entirely online.

## 5 Conclusion

This study found that Bedtime Window, a self-guided interactive digital CBTi program tailored for insomnia-alone and COMISA, was associated with large and sustained improvements in sleep, depression, anxiety, sleepiness, fatigue, and maladaptive beliefs about sleep in people with insomnia-alone and those with suspected COMISA. Given the high prevalence and adverse health consequences of COMISA, and extremely limited access to CBTi, these preliminary results highlight the potential to investigate the effectiveness, safety and acceptability of this digital CBTi program in people with a confirmed diagnosis of OSA in different settings, before scaling access to people with COMISA throughout the health system.

## Data availability statement

The datasets presented in this article are not readily available because of Human Research Ethics Committee requirements. Requests to access the datasets should be directed to AS, alexander.sweetman@flinders.edu.au.

## Ethics statement

The studies involving humans were approved by the Southern Adelaide Clinical Human Research Ethics Committee (2021/HRE00287). The studies were conducted in accordance with the local legislation and institutional requirements. The participants provided their written informed consent to participate in this study.

## Author contributions

AS: Conceptualization, Data curation, Formal analysis, Investigation, Methodology, Project administration, Resources, Visualization, Writing – original draft, Writing – review & editing. CRe: Investigation, Methodology, Project administration, Resources, Writing – review & editing. LL: Writing – review & editing. AV: Writing – review & editing. CC-C: Writing – review & editing. DW: Writing – review & editing. MC: Writing – review & editing. CRi: Data curation, Investigation, Methodology, Project administration, Resources, Writing – review & editing.

## References

[B1] AASM (2014). The American Academy of Sleep Medicine, 3rd Edn. Westchester, IL.

[B2] Alessi C. Fung C. Dzierzewski J. Fiorentino L. Stepnowsky C. Rodriguez Tapia J. C.. (2021). Randomized controlled trial of an integrated approach to treating insomnia and improving use of positive airway pressure therapy in veterans with comorbid insomnia disorder and obstructive sleep apnea. Sleep 44:zsaa235. 10.1093/sleep/zsaa235 33221910 PMC8033453

[B3] ASA (2024). Sleep Heatlh Primary Care Resource: Evidence-Based Resources and Information to Assess and Manage Adult Patients With Obstructive Sleep apnea and Insomnia. Available online at: https://www.sleepprimarycareresources.org.au/ (accessed February, 2024).

[B4] Baglioni C. Altena E. Bjorvatn B. Blom K. Bothelius K. Devoto A.. (2020). The European Academy for Cognitive Behavioural Therapy for Insomnia: an initiative of the European Insomnia Network to promote implementation and dissemination of treatment. J. Sleep Res. 29:e12967. 10.1111/jsr.12967 31856367

[B5] Bastien C. Vallières A. (2001). Validation of the insomnia severity index as an outcome measure for insomnia research. Sleep Med. 2, 297–307. 10.1016/S1389-9457(00)00065-4 11438246

[B6] Bianchi M. Williams K. L. McKinney S. Ellenbogen J. M. (2013). The subjective-objective mismatch in sleep perception among those with insomnia and sleep apnea. J. Sleep Res. 22, 557–568. 10.1111/jsr.12046 23521019

[B7] Björnsdóttir E. Keenan B. T. Eysteinsdottir B. Arnardottir E. S. Janson C. Gislason T.. (2015). Quality of life among untreated sleep apnea patients compared with the general population and changes after treatment with positive airway pressure. J. Sleep Res. 24, 328–338. 10.1111/jsr.12262 25431105 PMC4439289

[B8] Bjorvatn B. Berge T. Lehmann S. Pallesen S. Saxvig I. (2018). No effect of a self-help book for insomnia in patients with obstructive sleep apnea and comorbid chronic insomnia–a randomized controlled trial. Front. Psychol. 9:2413. 10.3389/fpsyg.2018.02413 30555398 PMC6281758

[B9] Bootzin R. R. (1972). Stimulus control for the treatment of insomnia. Proc. Am. Psychol. Assoc. 7, 395–396.

[B10] Chai-Coetzer C. L. Antic N. A. Rowland L. S. Catcheside P. G. Esterman A. Reed R. L.. (2011). A simplified model of screening questionnaire and home monitoring for obstructive sleep apnea in primary care. Thorax 66, 213–219. 10.1136/thx.2010.152801 21252389

[B11] Christensen H. Batterham P. J. Gosling J. A. Ritterband L. M. Griffiths K. M. Thorndike F. P.. (2016). Effectiveness of an online insomnia program (SHUTi) for prevention of depressive episodes (the GoodNight Study): a randomised controlled trial. Lancet Psychiatry 3, 333–341. 10.1016/S2215-0366(15)00536-2 26827250

[B12] Chung F. Abdullah H. R. Liao P. (2016). STOP-Bang questionnaire: a practical approach to screen for obstructive sleep apnea. Chest 149, 631–638. 10.1378/chest.15-0903 26378880

[B13] Eldridge-Smith E. D. Manber R. Tsai S. Kushida C. Simmons B. Johnson R.. (2022). Stepped care management of insomnia co-occurring with sleep apnea: the AIR study protocol. Trials 23, 1–12. 10.1186/s13063-022-06753-4 36153634 PMC9509569

[B14] Epstein L. J. Kristo D. Strollo P. J. Friedman N. Malhotra A. Patil S. P.. (2009). Clinical guideline for the evaluation, management and long-term care of obstructive sleep apnea in adults: adult obstructive sleep apnea task force of the American Academy of Sleep Medicine. J. Clin. Sleep Med. 5, 263–276. 10.5664/jcsm.27497 19960649 PMC2699173

[B15] Espie C. A. Emsley R. Kyle S. D. Gordon C. Drake C. L. Siriwardena A. N.. (2019). Effect of digital cognitive behavioral therapy for insomnia on health, psychological well-being, and sleep-related quality of life: a randomized clinical trial. JAMA Psychiatry 76, 21–30. 10.1001/jamapsychiatry.2018.2745 30264137 PMC6583463

[B16] Espie C. A. Kyle S. D. Williams C. Ong J. C. Douglas N. J. Hames P.. (2012). A randomized, placebo-controlled trial of online cognitive behavioral therapy for chronic insomnia disorder delivered via an automated media-rich web application. Sleep 35, 769–781. 10.5665/sleep.1872 22654196 PMC3353040

[B17] Gradisar M. Lack L. Richards H. Harris J. Gallasch J. Boundy M.. (2007). The Flinders Fatigue Scale: preliminary psychometric properties and clinical sensitivity of a new scale for measuring daytime fatigue associated with insomnia. J. Clin. Sleep Med. 3, 722–728. 10.5664/jcsm.27030 18198807 PMC2556916

[B18] Haycock J. Lack L. Hoon E. Sweetman A. Appleton S. Reynolds A. C.. (2022). O048 Help seeking behaviours of Australian adults with insomnia in a community sample. Sleep 3(Suppl. 1):A20. 10.1093/sleepadvances/zpac029.047

[B19] Johns M. W. (1991). A new method for measuring daytime sleepiness: The Epworth Sleepiness Scale. Sleep 14, 540–545. 10.1093/sleep/14.6.540 1798888

[B20] Krakow B. Melendrez D. Ferreira E. Clark J. Warner T. D. Sisley B.. (2001). Prevalence of insomnia symptoms in patients with sleep-disordered breathing. Chest 120, 1923–1929. 10.1378/chest.120.6.1923 11742923

[B21] Kroenke K. Spitzer R. L. Williams J. B. (2003). The Patient Health Questionnaire-2: validity of a two-item depression screener. Med. Care. 41, 1284–1292. 10.1097/01.MLR.0000093487.78664.3C 14583691

[B22] Kyle S. D. Miller C. B. Rogers Z. Siriwardena A. N. MacMahon K. M. Espie C. A.. (2014). Sleep restriction therapy for insomnia is associated with reduced objective total sleep time, increased daytime somnolence, and objectively-impaired vigilance: Implications for the clinical management of insomnia disorder. Sleep 37, 229–237. 10.5665/sleep.3386 24497651 PMC3900612

[B23] Lang C. J. Appleton S. L. Vakulin A. McEvoy R. D. Wittert G. A. Martin S. A.. (2017). Co-morbid OSA and insomnia increases depression prevalence and severity in men. Respirology 22, 1407–1415. 10.1111/resp.13064 28589663

[B24] Lechat B. Appleton S. Melaku Y. A. Hansen K. McEvoy R. D. Adams R.. (2022a). The association of co-morbid insomnia and obstructive sleep apnea with prevalent cardiovascular disease and incident cardiovascular events. J. Sleep Res. 31:e13563. 10.1111/jsr.13563 35166401

[B25] Lechat B. Loffler K. A. Wallace D. M. Reynolds A. Appleton S. L. Scott H.. (2021). Co-morbid insomnia and obstructive sleep apnea is associated with all-cause mortality. Euro Resp. J. 69:2101958. 10.1183/13993003.01958-2021

[B26] Lechat B. Loffler K. A. Wallace D. M. Reynolds A. Appleton S. L. Scott H.. (2022b). All-cause mortality in people with co-occurring insomnia symptoms and sleep apnea: analysis of the Wisconsin Sleep Cohort. Nat. Sci. Sleep 14, 1817–1828. 10.2147/NSS.S379252 36263373 PMC9576322

[B27] Lichstein K. Riedel B. Lester K. Aguillard R. (1999). Occult sleep apnea in a recruited sample of older adults with insomnia. J. Consult. Clin. Psychol. 67, 405–410. 10.1037/0022-006X.67.3.405 10369061

[B28] Luyster F. S. Buysse D. J. Strollo P. J. (2010). Comorbid insomnia and obstructive sleep apnea: challenges for clinical practice and research. J. Clin. Sleep Med. 6:27772. 10.5664/jcsm.27772 20411700 PMC2854710

[B29] Miller C. B. Valenti L. Harrison C. M. Bartlett D. J. Glozier N. Cross N. E.. (2017). Time trends in the family physician management of insomnia: the Australian experience (2000–2015). J. Clin. Sleep Med. 13, 785–790. 10.5664/jcsm.6616 28454597 PMC5443739

[B30] Morin C. M. Culbert J. P. Schwartz S. M. (1994). Nonpharmacological interventions for insomnia: a meta-analysis of treatment efficacy. Am. J. Psychiatry. 151, 1172–1180. 10.1176/ajp.151.8.1172 8037252

[B31] Morin C. M. Vallières A. (2007). Dysfunctional beliefs and attitudes about sleep (DBAS): validation of a brief version (DBAS-16). Sleep 30, 1547–1554. 10.1093/sleep/30.11.1547 18041487 PMC2082102

[B32] Nazem S. Barnes S. M. Forster J. E. Hostetter T. A. Monteith L. L. Kramer E. B.. (2023). Efficacy of an internet-delivered intervention for improving insomnia severity and functioning in veterans: randomized controlled trial. JMIR Mental Health 10:e50516. 10.2196/50516 37999953 PMC10709797

[B33] Ong J. C. Crawford M. R. Dawson S. C.. (2020b). A randomized controlled trial of CBT-I and PAP for obstructive sleep apnea and comorbid insomnia: main outcomes from the MATRICS study. Sleep 43:zsaa041. 10.1093/sleep/zsaa041 32170307 PMC7487869

[B34] Ong J. C. Crawford M. R. Kong A. Park M. Cvengros J. A. Crisostomo M. I.. (2017). Management of obstructive sleep apnea and comorbid insomnia: a mixed-methods evaluation. Behav. Sleep Med. 15, 180–197. 10.1080/15402002.2015.1087000 26670949

[B35] Ong J. C. Crawford M. R. Wallace D. M. (2020a). Sleep apnea and insomnia: emerging evidence for effective clinical management. Chest 159, 2020–2028. 10.1016/j.chest.2020.12.002 33309524 PMC8129729

[B36] Pfeiffer P. N. Ganoczy D. Zivin K. Gerlach L. Damschroder L. Ulmer C. S.. (2023). Guideline-concordant use of cognitive behavioral therapy for insomnia in the Veterans Health Administration. Sleep Health 9, 893–896. 10.1016/j.sleh.2023.07.002 37704561

[B37] Qaseem A. Kansagara D. Forciea M. A. Cooke M. Denberg T. D. (2016). Management of chronic insomnia disorder in adults: a clinical practice guideline from the American College of Physicians. Ann. Intern. Med. 165, 125–133. 10.7326/M15-2175 27136449

[B38] Ree M. Junge M. Cunnington D. (2017). Australasian Sleep Association position statement regarding the use of psychological/behavioral treatments in the management of insomnia in adults. Sleep Med. 36, S43–S47. 10.1016/j.sleep.2017.03.017 28648226

[B39] Ritterband L. M. Thorndike F. P. Ingersoll K. S. Lord H. R. Gonder-Frederick L. Frederick C.. (2017). Effect of a web-based cognitive behavior therapy for insomnia intervention with 1-year follow-up: a randomized clinical trial. JAMA Psychiatry 74, 68–75. 10.1001/jamapsychiatry.2016.3249 27902836

[B40] Sivertsen B. Björnsdóttir E. Øverland S. Bjorvatn B. Salo P. (2013). The joint contribution of insomnia and obstructive sleep apnea on sickness absence. J. Sleep Res. 22, 223–230. 10.1111/j.1365-2869.2012.01055.x 23043357

[B41] Soh H. L. Ho R. C. Ho C. S. Tam W. W. (2020). Efficacy of digital cognitive behavioural therapy for insomnia: a meta-analysis of randomised controlled trials. Sleep Med. 75, 315–325. 10.1016/j.sleep.2020.08.020 32950013

[B42] Spielman A. J. Saskin P. (1987). Treatment of chronic insomnia by restriction of time in bed. Sleep 10, 45–56. 3563247

[B43] Spitzer R. L. Kroenke K. Williams J. B. Löwe B. (2006). A brief measure for assessing generalized anxiety disorder: the GAD-7. Arch. Intern. Med. 166, 1092–1097. 10.1001/archinte.166.10.1092 16717171

[B44] Sweetman A. (2022a). Digital Insomnia Treatment in Australian Primary Care. ANZCTR. Available online at: https://anzctr.org.au/Trial/Registration/TrialReview.aspx?id=384943andisReview=true (accessed February, 2024).

[B45] Sweetman A. (2022b). Effect of digital Cognitive Behavioural Therapy for Insomnia (dCBTi) in People With Co-Morbid Insomnia and Sleep apnea: A Randomised Waitlist Controlled Trial. Available online at: https://www.anzctr.org.au/Trial/Registration/TrialReview.aspx?id=384648andisReview=true (accessed February, 2024).

[B46] Sweetman A. Farrell S. Wallace D. Crawford M. (2023a). The effect of cognitive behavioural therapy for insomnia in people with co-morbid insomnia and sleep apnea: a systematic review and meta-analysis. J. Sleep Res. 32:e13847. 10.1111/jsr.13847 36872072

[B47] Sweetman A. Lack L. Bastien C. (2019a). Co-morbid insomnia and sleep apnea (COMISA): Prevalence, consequences, methodological considerations, and recent randomized controlled trials. Brain Sci. 9:371. 10.3390/brainsci9120371 31842520 PMC6956217

[B48] Sweetman A. Lack L. Catcheside P. G. Antic N. A. Smith S. Chai-Coetzer C. L.. (2019b). Cognitive and behavioral therapy for insomnia increases the use of continuous positive airway pressure therapy in obstructive sleep apnea participants with co-morbid insomnia: A randomized clinical trial. Sleep 42:zsz178. 10.1093/sleep/zsz178 31403168

[B49] Sweetman A. Lack L. Lambert S. Gradisar M. Harris J. (2017b). Does co-morbid obstructive sleep apnea impair the effectiveness of cognitive and behavioral therapy for insomnia? Sleep Med. 39, 38–46. 10.1016/j.sleep.2017.09.003 29157586

[B50] Sweetman A. Lack L. McEvoy D. Antic N. A. Smith S. Chai-Coetzer C. L.. (2020a). Cognitive behavioural therapy for insomnia reduces sleep apnea severity: a randomised controlled trial. ERJ OR. 6:00161-2020. 10.1183/23120541.00161-2020 32440518 PMC7231124

[B51] Sweetman A. Lack L. McEvoy R. Smith S. Eckert D. J. Osman A.. (2021). Bi-directional relationships between co-morbid insomnia and sleep apnea (COMISA). Sleep Med. Rev. 60:101519. 10.1016/j.smrv.2021.101519 34229295

[B52] Sweetman A. Lechat B. Appleton S. Reynolds A. Adams R. Melaku Y.. (2022). Association of co-morbid insomnia and sleep apnea symptoms with all cause mortality: analysis of the NHANES 2005-2008 data. Sleep Epidemiol. 2:100043. 10.1016/j.sleepe.2022.100043

[B53] Sweetman A. McEvoy R. D. Smith S. Catcheside P. G. Antic N. A. Chai-Coetzer C. L.. (2020b). The effect of cognitive and behavioral therapy for insomnia on week-to-week changes in sleepiness and sleep parameters in insomnia patients with co-morbid moderate and severe sleep apnea: a randomized controlled trial. Sleep 43:zsaa002. 10.1093/sleep/zsaa002 31927569

[B54] Sweetman A. Osman A. Lack L. Crawford D. Wallace M. (2023b). Co-morbid insomnia and sleep apnea (COMISA): recent research and future directions. Curr. Opin. Pulm. Med. 29, 567–573. 10.1097/MCP.0000000000001007 37642477

[B55] Sweetman A. Reynolds C. Richardson C. (2023c). Digital CBT-i versus digital sleep education control in an Australian community-based cohort: a randomised controlled trail. Sleep Adv. 4(Suppl. 1):A12. 10.1093/sleepadvances/zpad035.035 39257295

[B56] Sweetman A. M. Lack L. C. Catcheside P. G. Antic N. A. Chai-Coetzer C. L. Smith S. S.. (2017a). Developing a successful treatment for co-morbid insomnia and sleep apnea. Sleep Med. Rev. 33, 28–38. 10.1016/j.smrv.2016.04.004 27401786

[B57] Thorndike F. P. Saylor D. K. Bailey E. T.. (2008). Development and perceived utility and impact of an internet intervention for insomnia. Eur. J. Appl. Psychol. 4:32. 10.7790/ejap.v4i2.133 20953264 PMC2954428

[B58] Turner A. Ong J. Jones A. Tu A. Salanitro M. Crawford M.. (2023). Neurocognitive functioning in COMISA patients is better after PAP therapy, but worse after CBT-I: exploratory analysis of cognitive outcomes from the MATRICS study. Sleep. 46, 1–13. 10.31234/osf.io/4h8ne PMC10424173 37148183

[B59] Vakulin A. Baulk S. D. Catcheside P. G. Antic N. A. van den Heuvel C. J. Dorrian J.. (2009). Effects of alcohol and sleep restriction on simulated driving performance in untreated patients with obstructive sleep apnea. Ann. Intern. Med. 151, 447–455. 10.7326/0003-4819-151-7-200910060-00005 19805768

[B60] van der Zweerde T. Bisdounis L. Kyle S. D. Lancee J. van Straten A. (2019a). Cognitive behavioral therapy for insomnia: a meta-analysis of long-term effects in controlled studies. Sleep Med. Rev. 48:101208. 10.1016/j.smrv.2019.08.002 31491656

[B61] van der Zweerde T. van Straten A. Effting M. Kyle S. Lancee J. (2019b). Does online insomnia treatment reduce depressive symptoms? A randomized controlled trial in individuals with both insomnia and depressive symptoms. Psychol. Med. 49, 501–509. 10.1017/S0033291718001149 29747706 PMC6331685

[B62] Vedaa Ø. Kallestad H. Scott J. Smith O. R. F. Pallesen S. Morken G.. (2020). Effects of digital cognitive behavioural therapy for insomnia on insomnia severity: a large-scale randomised controlled trial. Lancet Digital Health 2, e397–e406. 10.1016/S2589-7500(20)30135-7 33328044

[B63] Wallace D. M. Wohlgemuth W. K. (2019). Predictors of insomnia severity index profiles in US veterans with obstructive sleep apnea. J. Clin. Sleep Med. 15, 1827–1837. 10.5664/jcsm.8094 31855168 PMC7099195

[B64] Weaver T. E. Grunstein R. R. (2008). Adherence to continuous positive airway pressure therapy: the challenge to effective treatment. Proc. Am. Thorac. Soc. 5, 173–178. 10.1513/pats.200708-119MG 18250209 PMC2645251

[B65] Wilson D. (2017). Practical Meta-Analysis Effect Size Calculator. Available online at: https://campbellcollaboration.org/research-resources/effect-size-calculator.html (accessed February, 2024).

[B66] Ye Y.-Y. Zhang Y.-F. Chen J. Liu J. Li X.-J. Liu Y.-Z.. (2015). Internet-based cognitive behavioral therapy for insomnia (ICBT-i) improves comorbid anxiety and depression—a meta-analysis of randomized controlled trials. PLoS ONE 10:e0142258. 10.1371/journal.pone.0142258 26581107 PMC4651423

